# A negative association between triglyceride glucose-body mass index and testosterone in adult males: a cross-sectional study

**DOI:** 10.3389/fendo.2023.1187212

**Published:** 2023-06-09

**Authors:** Shenghao Wu, Yanhong Wu, Lizi Fang, Junzhao Zhao, Yaoyao Cai, Weiting Xia

**Affiliations:** ^1^Reproductive Medicine Center, Department of Obstetrics and Gynecology, The Second Affiliated Hospital and Yuying Children’s Hospital of Wenzhou Medical University, Wenzhou, Zhejiang, China; ^2^Department of Obstetrics, The First Affiliated Hospital of Wenzhou Medical University, Wenzhou, Zhejiang, China; ^3^Department of Gynecology, The First Affiliated Hospital of Wenzhou Medical University, Wenzhou, Zhejiang, China

**Keywords:** testosterone, triglyceride glucose-body mass index, Insulin resistance, NHANES, TyG-BMI

## Abstract

**Background and objectives:**

Insulin resistance (IR) is closely related to the decline or deficiency of testosterone in males. Triglyceride glucose-body mass (TyG-BMI) is considered to be a novel indicator of IR. We conducted this analysis to investigate the association between TyG-BMI and male testosterone, and to explore whether its ability to predict testosterone deficiency is superior to HOMA-IR and TyG.

**Methods:**

This was a cross-sectional study using data from the National Health and Nutrition Examination Survey (NHANES, 2011–2016). The TyG-BMI index was calculated from serum triglyceride, fasting plasma glucose and BMI. The association of TyG-BMI with male testosterone was estimated by weighted multivariable regression.

**Results:**

We included 3394 participants for the final analysis. After adjusting for confounders, TyG-BMI was found to show an independent negative association with testosterone (β=-1.12, 95%CI: -1.50, -0.75, P<0.0001). Multivariate-adjusted beta also showed testosterone levels were significantly lower in the two highest TyG-BMI group (Q3, Q4) compared to the lowest group (Q1). Similar results were seen in all of the subgroup populations by stratified analysis (all P-interaction >0.05). Furthermore, ROC curve analysis indicated that the area under the curve of TyG-BMI index (0.73, 95% CI: 0.71, 0.75) was larger than that of HOMA-IR index (0.71, 95% CI: 0.69, 0.73) and TyG index (0.66, 95% CI: 0.64, 0.68).

**Conclusion:**

Our result suggested a negative association between TyG-BMI index and testosterone in adult males. The predictability of the TyG-BMI index for testosterone deficiency is better than that of HOMA-IR index and TyG index.

## Introduction

Testosterone is the main male sex hormone secreted by Leydig cells. A normal testosterone level is essential in male physiological processes, including sexual function, cardiovascular health, metabolism, cerebral function, and bone density (1–3). The decrease or deficiency of serum testosterone levels in males may cause dysfunction in multiple organs. In addition to decreased libido and erectile dysfunction, low testosterone level can also cause or aggravate relative metabolic diseases, such as depression and osteoporosis, the so-called testosterone deficiency syndrome (4–7), or male hypogonadism (8). Testosterone deficiency is a common disorder that affects about 7% of males over 50s, and the incidence increases with age. It is estimated that this condition will increase with the average life span in the following decades (9). Testosterone deficiency has become an increasingly alarming issue worldwide.

Insulin Resistance (IR) is an essential influence factor affecting the occurrence and progression of cardiometabolic diseases. And hypogonadism has been found to be common in metabolic comorbidities, such as obesity and diabetes (10). In this case, several studies highlight that IR is closely associated with deficiency of testosterone. Souteiro et al. (11) found IR is the primary risk factor of low testosterone levels in obese men, and some males with testosterone deficiency even have a higher IR index than patients with mild diabetes. Another pilot study found that Metformin, a diabetes drug, can restore the testosterone level of male patients with type II diabetes through improved IR (12). On the other hand, the decrease or deficiency of testosterone may lead to the development of metabolic disorders through increased visceral lipids and insulin resistance (13). Furthermore, in functional hypogonadism as well as in late-onset hypogonadism, the relationship between hypogonadism and metabolic disorders has been proven to be bidirectional (14). Therefore, the correlation of IR with male testosterone is becoming a research hotspot.

Traditional IR evaluations are complicated, time-consuming, and with limited application in the research environment, including hyperinsulinemic-euglycemic clamp (HIEC) and homeostasis model assessment (HOMA-IR). The triglyceride-glucose index (TyG index), first brought up by South American researchers, has a good correlation with HIEC and HOMA-IR index. This index is easy to obtain and less costly than traditional IR evaluations (15). Based on the TyG index, the triglyceride-glucose-body mass index (TyG-BMI) was officially proposed in 2016. It has improved the predictability by including with BMI, one of the obesity indicators. Er, et al. (16) found that TyG-BMI has the strongest association with HOMA-IR compared to various biomarkers and believed it has more diagnostic significance than the TyG index. In recent years, TyG-BMI has been approved as effective in the evaluation of atherosclerosis, prehypertension, and prediabetes, and thus was becoming a new area of research (17). However, the association between TyG-BMI and testosterone remains unclear, so is a higher TyG-BMI index associated with decreased testosterone levels in adult males?

Therefore, this study aims to explore the association between the TyG-BMI and male testosterone among a nationally representative sample of U.S. adults, and to access the predictability of the TyG-BMI index for testosterone deficiency, hoping to provide new perspectives on male reproductive health.

## Materials and methods

### Subjects

This study was a cross-sectional study. Data were obtained from the National Health and Nutrition Examination Survey (NHANES), a research program sponsored by the Centers for Disease Control and Prevention (CDC) to assess the health condition of U.S. citizens between 2011 and 2016. A total of 14751 U.S. males participated in NHANES, and the data covered demography, socioeconomics, diet, and health. Physical examination included physiological measurements, laboratory examination. Additionally, the samples were highly representative of the national population of US as NHANES applied a stratified multi-stage sampling method. All the data we used from NHANES could be obtained from the website (https://www.cdc.gov/nchs/nhanes/). The study of NHANES was approved by the Research Ethics Review Board of the National Center for Health Statistics (NCHS).

### Data collection and definition

This study used a standard questionnaire to collect demographic data, including age, race, marital status, education level, household income, smoking status, drinking status, and personal medical history (hypertension, diabetes, and sleeping disorder). The poverty income ratio (RIP) used as a proxy measure of household economic status. Drinking status was defined based on daily alcohol consumption, ≤1 drinks per week was a non or a light drinker, 2-8 was a moderate drinker, and >8 was a heavy drinker. The body measures data including body mass index (BMI) and waist circumference (WC) were collected at the Mobile Examination Center (MEC). The participants were divided into three groups based on BMI, normal or underweight: BMI <25 kg/m^2^, overweight: BMI between 25 and 29.9 kg/m^2^, and obese: BMI ≥30 kg/m^2^. Blood collection was performed in the morning after fasting to collect total cholesterol, high-density lipoprotein (HDL), low-density lipoprotein (LDL), triglyceride, fasting blood glucose, insulin and testosterone. Hypertension and diabetes were obtained from the self-report of health questionnaire, the investigators asked the question “Ever told you by doctor have diabetes?” or “Ever told you by doctor have high blood pressure?”. An answer of “yes” indicates a “diabetes” case or “hypertension” case. According to the American Urological Association guidelines (18), testosterone deficiency was defined as total testosterone <300 ng/dL.

BMI calculation: BMI=body weight (kg)/height (m)^2^;

TyG index calculation: TyG=Ln[Fasting triglycerides (mg/dL)×fasting plasma glucose (mg/dL)/2] (19);

TyG-BMI index calculation: TyG-BMI=TyG index×BMI (kg/m^2^) (16);

HOMA-IR index calculation: HOMA-IR=fasting plasma glucose (mmol/L)×fasting insulin (μU/mL)/22.5 (20);

TyG-WC index calculation: TyG-BMI=TyG index×WC (cm) (21).

### Statistical analysis

Continuous variables described by a normal distribution were presented as Mean ± Standard Deviation (M ± SD). Non-normally distributed data were presented as median (interquartile range). Categorical variables were presented with numbers (n) and percentages (%). The Chi-square test or Kruskal-Wallis H test was used for quartile groups of different TyG-BMI indexs. Regression model analysis was used to assess the association between the TyG-BMI and testosterone, using β values and 95% confidence intervals indicating the relationship. We applied three models, model I with adjusting none, model II adjusted age and race, and model III adjusted age, race, marital status, household income, BMI status, smoking status, drinking status, triglycerides, fasting blood glucose, hypertension, diabetes, sleep disorders. We then used restricted cubic spline curves based on regression model III to explore any non-linear relationship between the TyG-BMI index and testosterone.

Further, stratified analyses were conducted according to age, race, marital status, education level, household income, BMI status, smoking status, drinking status, and histories of chronic diseases. Their interactions were tested by log-likelihood ratio test.

At last, the ability of the TyG index, TyG-BMI index, TyG-WC index and HOMA-IR index to predict testosterone deficiency was analyzed by the receiver operating characteristic (ROC) curve, and the area under the ROC curve (AUROC) was calculated. Z test was applied to test differences between AUROCs.

We applied weighted analysis to explain the complex sampling of NHANES. Details can be found on this website (https://wwwn.cdc.gov/nchs/Thestatisticalsoftwareusedbynhanes/tutorials/module3.aspx). R language 4.2.1 was used to analyze all data. In all analyses, p <0.05 were considered statistically significant.

## Results

### Baseline characteristics of participants based on TyG-BMI index

**Figure 1:**
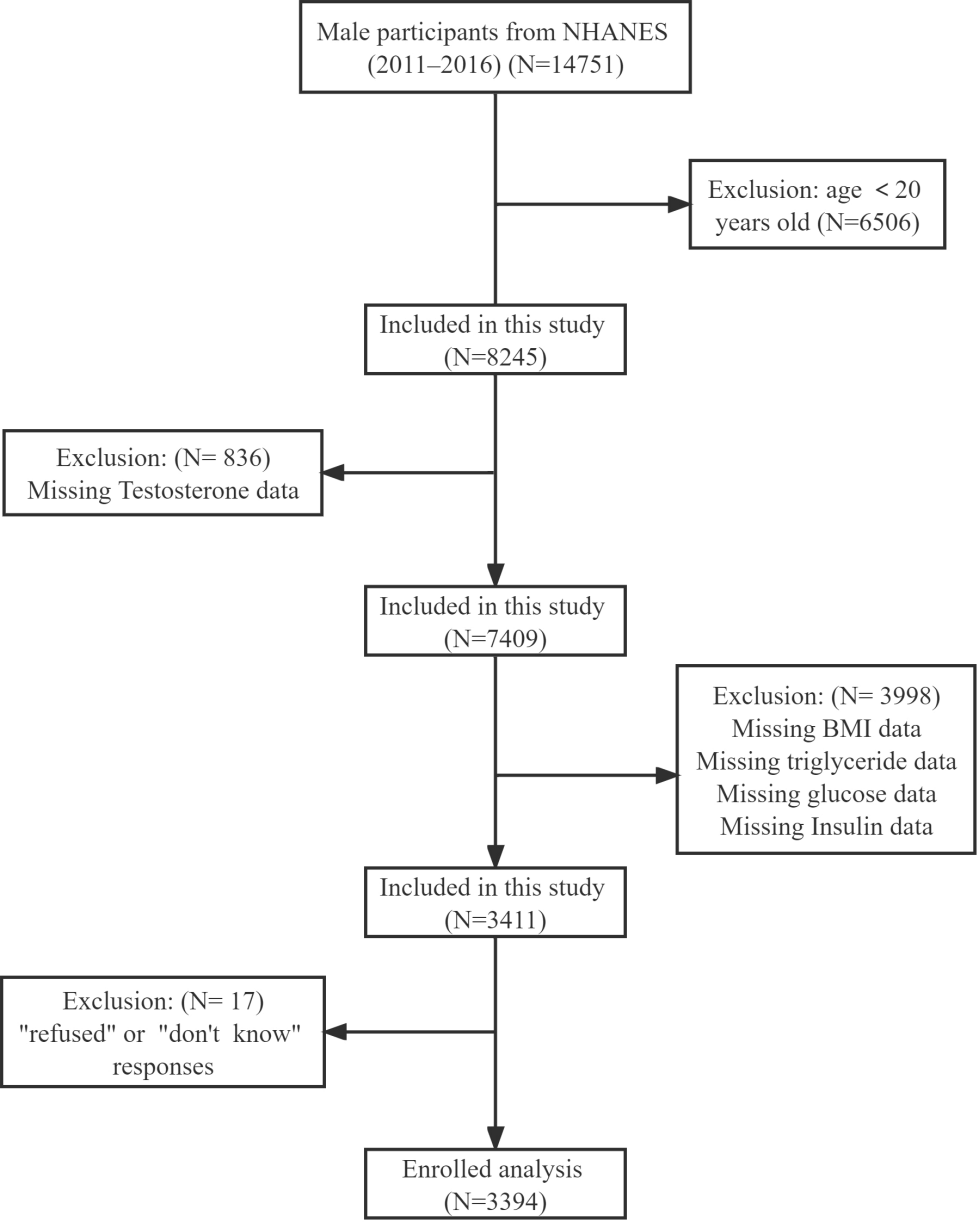
Flow chart of the screening process for the selection of eligible participants.

The data came from an NHANES program including 14751 male participants from 2011-2016. In our study, we included participants aged over 20 years. Participants were excluded with missing testosterone, BMI, triglyceride, and fasting blood glucose data, Additionally, anyone who responded with “refuse” or “not clear” to the question “Ever told you by doctor have high blood pressure, diabetes or sleep disorder” was excluded. After that, we included 3394 participants for the final analysis (**Figure 1**). We divided participants into four groups according to TyG-BMI quartiles: Q1≤ 205.65, 205.65<Q2 ≤ 239.59, 239.59<Q3 ≤ 280.19, and Q4>280.19. The baseline characteristics of each group were shown in **Table 1**. We found that Q4 participants had the highest HOMA-IR index (9.38 ± 17.89) and lowest testosterone level (337.99 ± 158.21). At the same time, they were more likely to be obese (92.90%), and with complications such like hypertension (51.01%), diabetes (25.33%), and sleeping disorders (26.27%). All variables, except education level and drinking status, were significantly different among the four groups (all p<0.05).

**Table 1:** Demographic and clinical characteristics according to triglyceride glucose-body mass index level.

TyG-BMI index quartile	Total	Q1	Q2	Q3	Q4	P-value
**Number**	3394	850	847	852	845	
**Age (years) group**						<0.001
20-39	1127 (33.21%)	393 (46.24%)	242 (28.57%)	248 (29.11%)	244 (28.88%)	
40-59	1133 (33.38%)	210 (24.71%)	289 (34.12%)	311 (36.50%)	323 (38.22%)	
≥60	1134 (33.41%)	247 (29.06%)	316 (37.31%)	293 (34.39%)	278 (32.90%)	
**Race**						<0.001
Mexican American	452 (13.32%)	64 (7.53%)	93 (10.98%)	148 (17.37%)	147 (17.40%)	
Other Hispanic	360 (10.61%)	67 (7.88%)	85 (10.04%)	115 (13.50%)	93 (11.01%)	
Non-Hispanic White	1387 (40.87%)	344 (40.47%)	328 (38.72%)	334 (39.20%)	381 (45.09%)	
Non-Hispanic Black	651 (19.18%)	169 (19.88%)	181 (21.37%)	133 (15.61%)	168 (19.88%)	
Other Race	544 (16.03%)	206 (24.24%)	160 (18.89%)	122 (14.32%)	56 (6.63%)	
**Marital status**						<0.001
Married	1924 (56.69%)	381 (44.82%)	505 (59.62%)	518 (60.80%)	520 (61.54%)	
Other	1470 (43.31%)	469 (55.18%)	342 (40.38%)	334 (39.20%)	325 (38.46%)	
**Education level**						0.769
Less than high school	818 (24.10%)	206 (24.24%)	193 (22.79%)	210 (24.65%)	209 (24.73%)	
High school or above	2576 (75.90%)	644 (75.76%)	654 (77.21%)	642 (75.35%)	636 (75.27%)	
**Household income**						0.015
0–1.3RIP	1003 (32.22%)	272 (35.10%)	221 (28.55%)	247 (31.55%)	263 (33.67%)	
> 1.3–3.5 RIP	1131 (36.33%)	283 (36.52%)	271 (35.01%)	297 (37.93%)	280 (35.85%)	
> 3.5 RIP	979 (31.45%)	220 (28.39%)	282 (36.43%)	239 (30.52%)	238 (30.47%)	
**BMI status**						<0.001
Normal or low weight	987 (29.08%)	775 (91.18%)	198 (23.38%)	14 (1.64%)	0 (0.00%)	
Overweight	1281 (37.74%)	75 (8.82%)	627 (74.03%)	519 (60.92%)	60 (7.10%)	
Obesity	1126 (33.18%)	0 (0.00%)	22 (2.60%)	319 (37.44%)	785 (92.90%)	
**Waist circumference (cm)**	102.07 ± 16.18	84.58 ± 7.79	96.61 ± 6.93	104.30 ± 7.31	120.63 ± 13.69	<0.001
**Smoking status**						<0.001
Every day	633 (34.36%)	207 (45.70%)	152 (33.26%)	149 (31.11%)	125 (27.59%)	
Some days	173 (9.39%)	45 (9.93%)	46 (10.07%)	44 (9.19%)	38 (8.39%)	
Not at all	1036 (56.24%)	201 (44.37%)	259 (56.67%)	286 (59.71%)	290 (64.02%)	
**Drinking status**						0.184
None or light drinker	645 (27.61%)	164 (28.42%)	175 (29.07%)	156 (26.94%)	150 (25.95%)	
Moderate drinker	1565 (66.99%)	388 (67.24%)	402 (66.78%)	390 (67.36%)	385 (66.61%)	
Heavy drinker	126 (5.39%)	25 (4.33%)	25 (4.15%)	33 (5.70%)	43 (7.44%)	
**Total cholesterol (mg/dL)**	186.68 ± 41.55	176.82 ± 38.05	185.68 ± 40.07	191.70 ± 41.30	192.53 ± 44.67	<0.001
**HDL cholesterol (mg/dL)**	49.06 ± 14.48	58.04 ± 16.89	51.67 ± 13.54	45.24 ± 10.26	41.27 ± 10.12	<0.001
**LDL cholesterol (mg/dL)**	112.03 ± 35.83	103.52 ± 32.85	113.16 ± 35.13	117.46 ± 36.18	114.22 ± 37.70	<0.001
**Triglyceride (mg/dL)**	131.82 ± 111.72	76.40 ± 37.36	104.46 ± 53.86	146.39 ± 87.47	200.32 ± 171.46	<0.001
**Fasting blood glucose (mg/dL)**	112.50 ± 38.08	99.79 ± 20.21	106.96 ± 28.28	113.10 ± 33.67	130.25 ± 54.46	<0.001
**Insulin (uU/mL)**	14.08 ± 22.79	6.68 ± 6.27	9.40 ± 7.27	13.52 ± 11.55	26.77 ± 40.27	<0.001
**Testosterone (ng/dL)**	447.16 ± 194.99	556.07 ± 202.95	476.33 ± 192.00	417.77 ± 153.74	337.99 ± 158.21	<0.001
**HOMA-IR index**	4.37 ± 9.90	1.75 ± 3.32	2.52 ± 2.42	3.84 ± 4.56	9.38 ± 17.89	<0.001
**TyG index**	8.67 ± 0.70	8.13 ± 0.49	8.50 ± 0.50	8.86 ± 0.54	9.21 ± 0.72	<0.001
**TyG-WC index**	886.81 ± 179.23	682.12 ± 75.16	815.49 ± 59.27	917.68 ± 64.20	1106.27 ± 133.01	<0.001
**Hypertension**						<0.001
Yes	1243 (36.62%)	193 (22.71%)	288 (34.00%)	331 (38.85%)	431 (51.01%)	
No	2151 (63.38%)	657 (77.29%)	559 (66.00%)	521 (61.15%)	414 (48.99%)	
**Diabetes**						<0.001
Yes	478 (14.08%)	47 (5.53%)	91 (10.74%)	126 (14.79%)	214 (25.33%)	
No	2830 (83.38%)	790 (92.94%)	741 (87.49%)	704 (82.63%)	595 (70.41%)	
Borderline	86 (2.53%)	13 (1.53%)	15 (1.77%)	22 (2.58%)	36 (4.26%)	
**Sleep disorders**						<0.001
Yes	492 (14.50%)	75 (8.82%)	74 (8.74%)	121 (14.20%)	222 (26.27%)	
No	2902 (85.50%)	775 (91.18%)	773 (91.26%)	731 (85.80%)	623 (73.73%)	

Values are presented as mean ± standard deviation or n (%).

Q, quartile; RIP, ratio of family income to poverty; BMI, body mass index; HDL, high-density lipoprotein; LDL, low-density lipoprotein; HOMA-IR, homeostasis model assessment of insulin resistance; TyG, triglyceride glucose index; TyG-WC, triglyceride glucose-waist circumference.

### The association between TyG-BMI index and testosterone

**Figure 2:**
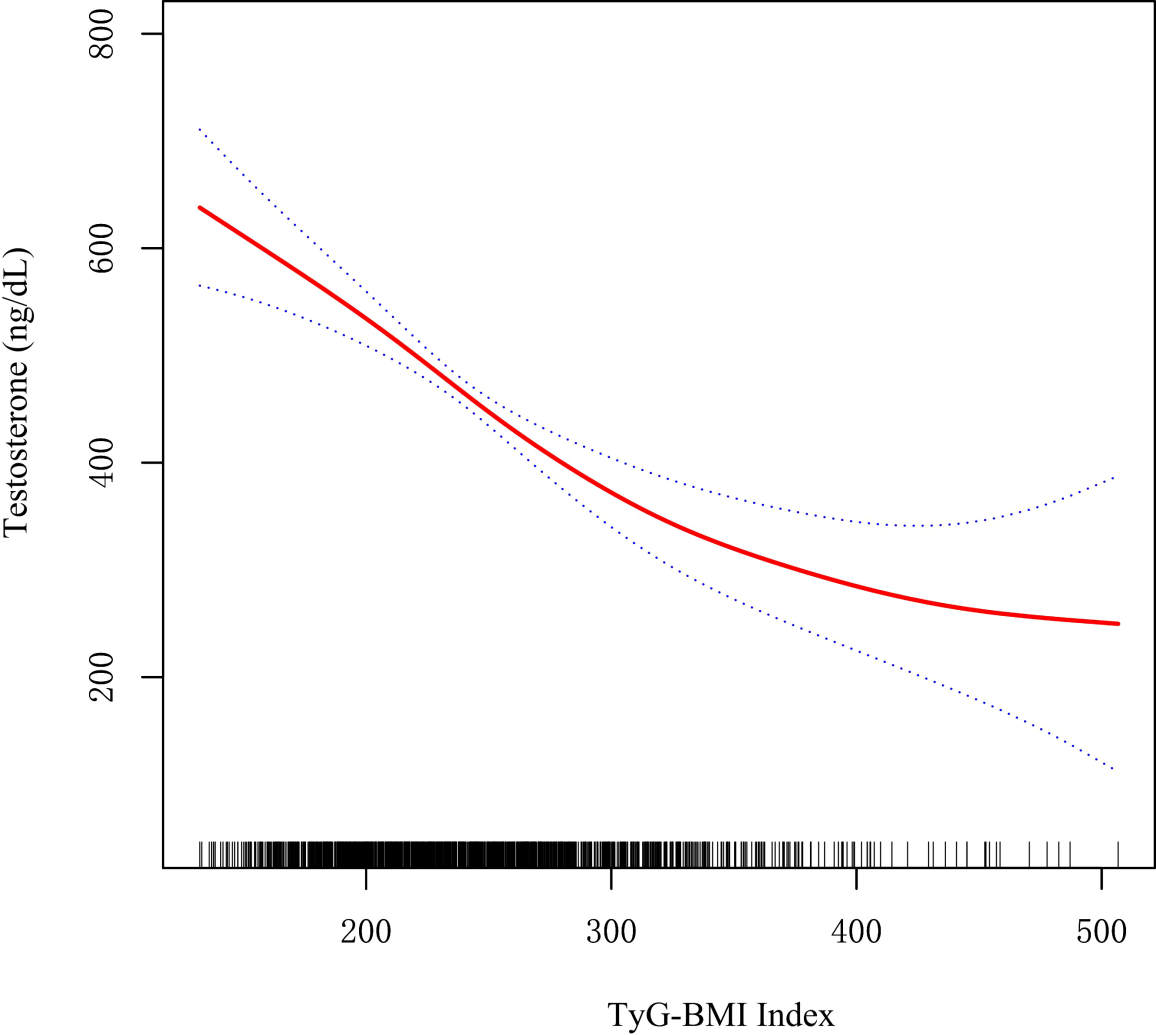
Restricted cubic spline fitting for the association between TyG-BMI index with testosterone levels.

As shown in **Figure 2**, restricted cubic spline (RCS) indicated a stable negative correlation between TyG-BMI and testosterone. **Table 2** showed the β values and 95% CI of TyG-BMI and testosterone in different models. The result suggested an independent negative correlation between TyG-BMI and testosterone in different adjusted models (model I, β= -1.34, 95% CI:-1.43, -1.25; model II, β= -1.33, 95% CI:-1.42, -1.24; model III, β= -1.12, 95% CI:-1.50, -0.75; all p < 0.0001). In Model I and II, the β values of the other three groups (Q2, Q3, Q4) were significantly different from that of Q1 (all p<0.05). In Model III, testosterone levels were significantly lower in the two highest TyG-BMI group (Q3, Q4) compared to the lowest group (Q1) after fully adjusting for confounders. In addition, the results of univariate regression analysis for testosterone were shown in **Table S1**.

**Table 2:** Multivariate regression analysis of triglyceride glucose-body mass index with testosterone.

Exposure	Model I β (95%CI) P-value	Model II β (95%CI) P-value	Model III β (95%CI) P-value
TyG-BMI index	-1.34 (-1.43, -1.25) <0.0001	-1.33 (-1.42, -1.24) <0.0001	-1.12 (-1.50, -0.75) <0.0001
TyG-BMI index quartile			
Q1	Ref.	Ref.	Ref.
Q2	-80.54 (-97.42, -63.66) <0.0001	-77.47 (-94.40, -60.53) <0.0001	-36.32 (-76.34, 3.70) 0.0756
Q3	-138.63 (-155.52, -121.75) <0.0001	-138.52 (-155.61, -121.44) <0.0001	-94.00 (-146.88, -41.12) 0.0005
Q4	-218.04 (-234.92, -201.15) <0.0001	-220.32 (-237.53, -203.10) <0.0001	-149.20 (-218.65, -79.75) <0.0001
P for trend	<0.0001	<0.0001	<0.0001

Model I adjust for: None.

Model II adjust for: Age, Race.

Model III adjust for: Age, Race, Marital status, Household income, BMI status, Smoking status, Drinking status, Triglyceride, Fasting blood glucose, Hypertension, Diabetes, Sleep disorders.

**Figure 3:**
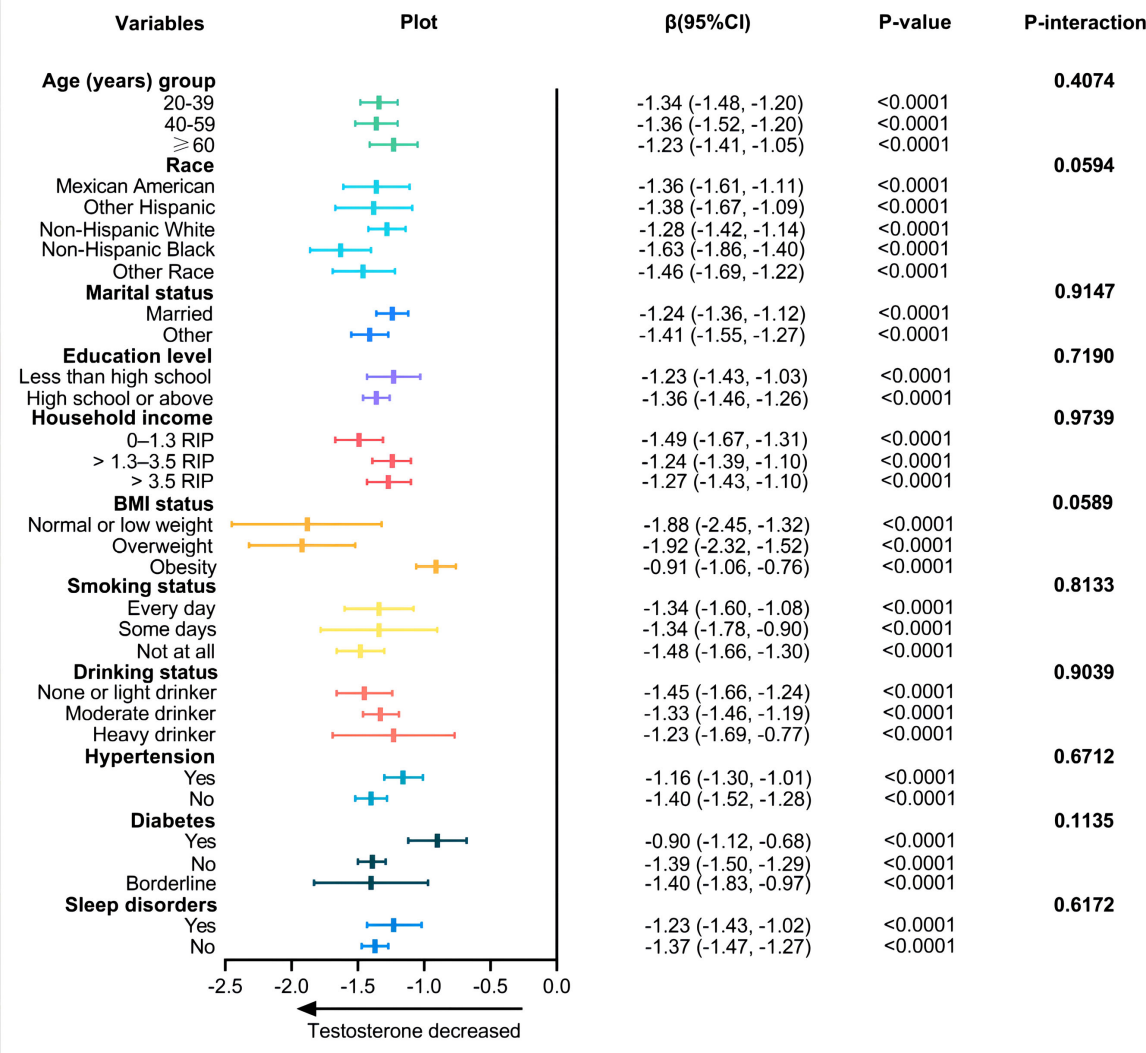
Associations between TyG-BMI index and testosterone according to baseline characteristics. The β-coefficients and 95% CI to the left of the plot are the numbers that are visualized in the plot. P-value is tested by linear regression analysis.

We used stratified analysis to assess the reliability of the correlation between TyG-BMI index and testosterone. And the results showed that the association of TyG-BMI with testosterone was similar in all sub-populations (all P-interaction >0.05, **Figure 3**).

### Comparison of different IR markers in predicting testosterone deficiency

**Figure 4:**
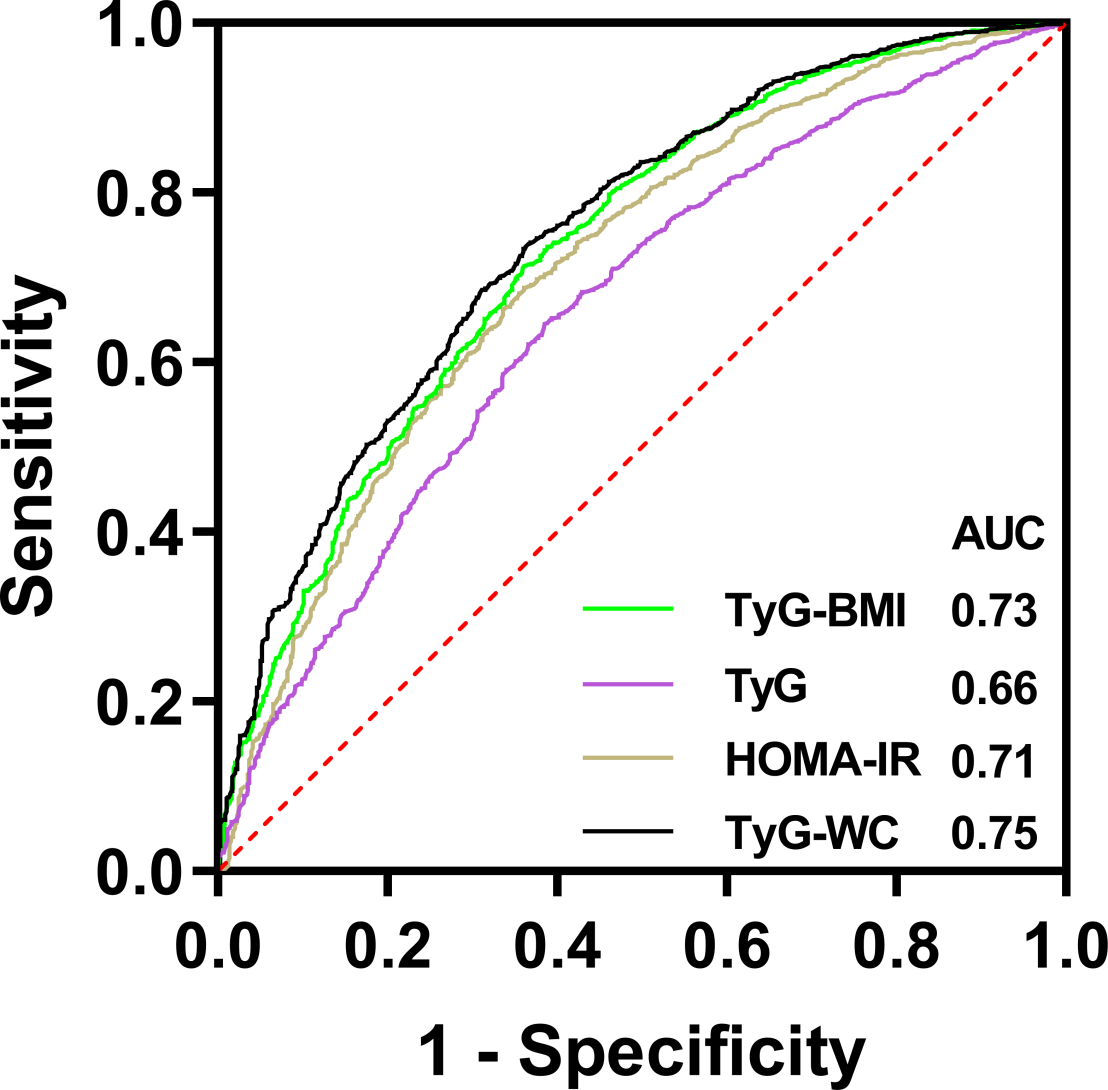
ROC curves for different insulin resistance markers to predict testosterone deficiency.

The result of ROC was shown in **Figure 4**. The AUROC of TyG index, TyG-BMI index, TyG-WC index and HOMA-IR index in predicting testosterone deficiency was 0.66 (95% CI: 0.64, 0.68), 0.73 (95% CI: 0.71, 0.75), 0.75 (95% CI: 0.73, 0.77) and 0.71 (95% CI: 0.69, 0.73), respectively. The results showed that TyG-BMI was similar to TyG-WC and better than TyG and HOMA-IR index in predicting testosterone deficiency for U.S. adult males, with a cutoff value of 259.9, a sensitivity and a specificity of 0.71 and 0.64, respectively (**Table 3**).

**Table 3:** Comparison of ROC curves for different insulin resistance markers to predict testosterone deficiency.

Markers	Cutoff (Sensitivity, Specificity)	AUC (95% CI)
TyG index	8.76 (0.65, 0.62)	0.66 (0.64, 0.68)
TyG-BMI index	259.9 (0.71, 0.64)	0.73 (0.71, 0.75)
HOMA-IR index	3.06 (0.66, 0.66)	0.71 (0.69, 0.73)
TyG-WC index	928.9 (0.74, 0.64)	0.75 (0.73, 0.77)

AUC, area under the curve; CI, confidence interval.

Z-test was used to compare statistical significant differences between AUCs.

## Discussion

This nationally representative cross-sectional study evaluated the relationship between TyG-BMI and testosterone levels. Our study suggested a negative correlation between high TyG-BMI and serum testosterone levels in U.S. adult males. The negative correlation remained stable after age, race, lifestyle, complications, and serological factors were adjusted. In addition, ROC analysis showed that the TyG-BMI index was better than TyG and HOMA-IR in predicting testosterone deficiency. To our knowledge, this was the first study exploring the relationship between TyG-BMI and testosterone in the general population.

The decrease or deficiency of testosterone in males is a growing concern because it is a critical factor leading to male sexual function disorders. Rastrelli et al. (22) found that decreased libido and erectile dysfunction in males were associated with low testosterone levels after collecting the clinical and biochemistry data from 3862 males with sexual function disorders. Another meta-analysis (23) from Africa including 17 studies, and 6002 participants showed that a testosterone level lower than 8 nmol/l is significantly associated with erectile dysfunction in diabetic patients. OU et al. (24) found that low male hormone may inhibit erection by up-regulating the expression of TRPC3, TRPC4, and TRPC6 in rat’s cavernous tissue. Current studies manifested that patients’ quality of life is seriously affected by testosterone deficiency because it accelerates the aging of body organs, as well as the development of systemic diseases such as cardiovascular diseases and Alzheimer (25, 26). Moreover, low testosterone may be associated with high mortality of cardiovascular diseases and type II diabetes (27–29). Additionally, low testosterone may also affect the prognosis of COVID-19 (30).

The common risk factors that may cause a decrease or deficiency in testosterone include advanced age, obesity, hyperlipidemia, and diabetes. IR is the critical physiological process in metabolic diseases, and is considered as an essential independent predictive factor of testosterone decrease or deficiency. Currently, the relationship between IR and testosterone deficiency has been proven to be bidirectional. IR may reduce the testosterone level, which will in turn promote obesity and IR (14). Tsai et al. (31) detected testosterone levels in 221 middle-aged males without diabetes, and the multivariate analysis result suggested a significant inverse relationship between testosterone and insulin, C-peptide and HOMA-IR index. A study that included 2361 Chinese males aged 20-73 showed that the total testosterone and metabolic syndrome remain negatively related (32). Similar results were found in a study targeted at males in Taiwan (33). A cross-sectional study by Liu et al. (34) showed that higher TyG index is associated with lower total testosterone level and higher risk of testosterone deficiency. However, their findings suggested that TyG index does not perform better than the HOMA-IR index in predicting testosterone deficiency.

The TyG-BMI index has been recommended as an alternative indicator of insulin resistance in recent years. Our result is similar to the findings of other diseases associated with IR. A study that evaluated the risk of atherosclerotic cardiovascular diseases (ASCVD) in 3143 Taiwanese adults found that TyG-BMI is highly associated with the increase in ASCVD risk (35). Another study of ischemic stroke (36) found that TyG-BMI has a linear correlation with ischemic stroke in the general population without threshold or saturation effects. For every standard deviation increase in TyG-BMI, the risk of ischemic stroke increased by 20%. Meanwhile, TyG-BMI is potentially practicable in improving risk stratification for ischemic stroke. Zeng et al. (37) performed a cross-sectional study that included 105070 non-obese adults with no hypertension and found that TyG-BMI and TyG-WC (waist circumference) index are significantly related to prehypertension. In addition, the OR of TyG-BMI and prehypertension was the highest. Furthermore, it was found that TyG-BMI is independently associated with non-small cell lung cancer (NSCLC) and osteoporotic fractures (38, 39).

However, the mechanism between TyG-BMI and male testosterone decrease remains unclear, which might be explained by the following points: Firstly, TyG-BMI is a composite index that contains glucose metabolism, blood lipid metabolism, and obese state. An abnormal index often signals hyperglycemia, hyperlipemia, and obesity. Insulin has been shown to maintain normal testosterone level by stimulating gonadotropin-releasing hormone (GnRH) expression nerves in the hypothalamus, which then promote GnRH secretion (40). While hyperglycemia may decrease the expression of mitochondrial acetylase 3, damaging mitochondrial function and insulin receptors in hypothalamic neurons. Thus, reducing the expression of GnRH genes and proteins in neurons, finally inhibiting GnRH neurons and causing a decrease in testosterone levels (41). Moreover, hyperglycemia can directly induce a decrease in Leydig’s cells-produced testosterone *via* activating the TLR4-mediated oxidative stress pathway (42). The effect of high glucose on the role of AQPs in Leydig cell steroidogenesis was analyzed in diabetic rats (*in-vivo*) and LC540 rat Leydig cells (*in vitro*) by Kannan et al. (43). They revealed that AQPs mediates the impairment of testicular steroidogenesis by hyperglycemia through the oxidative stress pathway. Secondly, since aromatase is expressed in adipocytes, its main role is to convert testosterone to estradiol in peripheral tissues. The increase in adipocytes in obese patients has increased the expression of aromatase, which ultimately lead to a higher peripheral conversion of testosterone (44). Secondly, triglycerides have been shown to have a negative correlation with testosterone levels. Sook et al. investigated the relationship between total testosterone and serum markers of metabolic syndrome in 8,606 Korean male workers and found that participants with low testosterone levels were more likely to have hypertriglyceridemia (45). Another high-quality meta-analysis from Brand et al. also suggested that low levels of testosterone were significantly associated with hypertriglyceridemia, and the magnitude of the association was greatest in non-overweight men (46). At last, obesity is also a common cause of testosterone decrease. Obesity-related leptin resistance mechanisms and inflammatory factors (including tumor necrosis factor-α, interleukin-1β, and C-reactive protein) mediate inflammatory responses suppress hypothalamic-pituitary-gonadal axis function in obese men, leading to decreased body testosterone levels (47, 48).

In our research, we found TyG-BMI is superior to TyG and HOMA-IR in predicting the development of testosterone deficiency in U.S. male adults. Our results are similar to the comparison of different IR markers in other diseases. A study from Korea (49) compared the association of the TyG index combined with the relative obesity index and IR by investigating 11149 participants. They found that the AUROC of IR predicted by TyG-BMI is the largest compared with other indexes. After conducting follow-up visits to over 100,000 participants with normal blood glucose, Jiang et al. (17) found the independent positive correlation (which is more obvious in non-obese, female, and younger patients) between TyG-BMI and prediabetes. They further compared the AUROC of TyG-BMI, TyG index, and BMI for predicting prediabetes and found that TyG-BMI has the largest AUROC. Another large population study based on 117056 participants (50) compared the association of the prevalence of hypertension with four IR substitute indexes, namely TyG, TyG-BMI, triglyceride/high-density lipoprotein cholesterol(TG/HDL-c), and metabolic score for IR (METS-IR). The result showed that TyG-BMI is the best for predicting hypertension compared to other biomarkers. Similarly, TyG-BMI shows excellence over other IR markers in predicting non-alcoholic fatty liver diseases and metabolic fatty liver diseases (51–53).

In this study, we applied weighted analysis to explain the complex sampling design of NHANES, making our conclusion more reliable and providing the basis for future multicenter cohort studies on testosterone deficiency and TyG-BMI. Since HOMA-IR is commonly affected by exogenous insulin injection in diabetes patients (54), we believed that TyG-BMI could replace other IR indexes in clinical settings as an effective one to evaluate the testosterone decrease or deficiency.

However, this study has some limitations. Firstly, we were unable to obtain gonadotropin data in this study due to the limitations of the NHANES database and therefore could not identify the type of hypogonadism. Secondly, this is a cross-sectional study, therefore causality cannot be obtained. The results can only be applied to the U.S. population instead of other regions. Thirdly, testosterone deficiency is not a simple biochemistry indicator. Other clinical conditions should be added into consideration. However, due to the limitations of the NHANES database, we could only define deficiency as total testosterone less than 300 ng/dL, and we were unable to obtain data on testicular volume and include it as a covariate in the study. Furthermore, this study only included adult males aged over 20, and a wider population should be further included. Finally, this study did not include data on drugs, such as the lipid-lowering drugs. Statins is one of the most commonly used lipid-lowering drugs, it may decrease serum levels of steroid hormones, including testosterone and cortisol, but the effects are considered to be quite slight (55, 56). At the same time, there are studies demonstrating that statins may not affect testosterone levels at all. A meta-analysis including six RCTs indicated that there was no difference between atorvastatin and placebo on total testosterone levels in men (57). Another cohort study showed that statins may reduce dehydroepiandrosterone (DHEA) and SHBG in males, but not total testosterone and bioavailable testosterone (58). Therefore, we suggest that further studies should incorporate these relevant factors.

## Conclusion

In a nationally representative sample of US adult males, there is a stable and strong negative correlation between TyG-BMI and testosterone in adult males. TyG-BMI is also better than TyG and HOMA-IR index in predicting the occurrence of testosterone deficiency in US adult males, but a wider population is needed to verify this relationship in future cohort studies.

## Data availability statement

The raw data supporting the conclusions of this article will be made available by the authors, without undue reservation.

## Ethics statement

All data from NHANES used in our analyses are publicly available at https://www.cdc.gov/nchs/nhanes/. The NHANES study was approved by the National Center for Health Statistics (NCHS) Research Ethics Review Board.

## Author contributions

SW and WX participated in the study design, wrote and modified the manuscript, and prepared tables and figures. YW, LF, and JZ were involved in the conduct of the study and data collection. SW, YC, and WX made contributions to data analysis and results interpretation. All authors contributed to the article and approved the submitted version.
