# Supplementary material for: A negative association between triglyceride glucose-body mass index and testosterone in adult males: a cross-sectional study
Source: Front Endocrinol (Lausanne). 2023 Jun 9;14:1187212. doi: 10.3389/fendo.2023.1187212 (PMC10289259; doi:10.3389/fendo.2023.1187212)
Supplement: Supplementary file 1 [file Table_1.docx]

**Table S1** Univariate logistic regression analysis of triglyceride glucose-body mass index with testosterone

| Variables | β(95%CI) | P-value |
| --- | --- | --- |
| **Age (years) group** |  |  |
| 20-39 | Ref. |  |
| 40-59 | -40.0 (-60.5, -19.6) | 0.0004 |
| ≥60 | -41.6 (-65.3, -17.8) | 0.0013 |
| **Race** |  |  |
| Mexican American | Ref. |  |
| Other Hispanic | -9.1 (-47.6, 29.4) | 0.6451 |
| Non-Hispanic White | -6.5 (-27.6, 14.6) | 0.5474 |
| Non-Hispanic Black | 24.4 (-1.7, 50.4) | 0.0736 |
| Other Race | 7.4 (-17.6, 32.4) | 0.5660 |
| **Marital status** |  |  |
| Married | Ref. |  |
| Other | 44.3 (27.7, 60.9) | <0.0001 |
| **Education level** |  |  |
| Less than high school | Ref. |  |
| High school or above | -7.6 (-31.2, 16.1) | 0.5333 |
| **Household income** |  |  |
| 0–1.3RIP | Ref. |  |
| > 1.3–3.5 RIP | -31.7 (-47.0, -16.4) | 0.0002 |
| > 3.5 RIP | -28.6 (-49.4, -7.8) | 0.0097 |
| **BMI status** |  |  |
| Normal or low weight | Ref. |  |
| Overweight | -101.8 (-124.1, -79.5) | <0.0001 |
| Obesity | -194.6 (-212.7, -176.6) | <0.0001 |
| **Waist circumference (cm)** | -5.20 (-5.69, -4.71) | <0.0001 |
| **Smoking status** |  |  |
| Every day | Ref. |  |
| Some days | -11.6 (-57.6, 34.3) | 0.6218 |
| Not at all | -56.6 (-83.0, -30.1) | 0.0001 |
| **Drinking status** |  |  |
| None or light drinker | Ref. |  |
| Moderate drinker | 9.9 (-14.7, 34.6) | 0.4327 |
| Heavy drinker | 58.2 (8.3, 108.1) | 0.0271 |
| **Total cholesterol (mg/dL)** | -0.1 (-0.3, 0.1) | 0.3110 |
| **HDL cholesterol (mg/dL)** | 3.3 (2.6, 4.0) | <0.0001 |
| **LDL cholesterol (mg/dL)** | 0.0 (-0.2, 0.2) | 0.9436 |
| **Triglyceride (mg/dL)** | -0.4 (-0.5, -0.3) | <0.0001 |
| **Fasting blood glucose (mg/dL)** | -1.0 (-1.3, -0.7) | <0.0001 |
| **Insulin (uU/mL)** | -2.1 (-3.3, -0.9) | 0.0019 |
| **HOMA-IR index** | -4.4 (-6.7, -2.2) | 0.0004 |
| **TyG index** | -88.2 (-99.5, -76.8) | <0.0001 |
| **TyG-WC index** | -0.50 (-0.53, -0.46) | <0.0001 |
| **TyG-BMI index** | -1.3 (-1.5, -1.2) | <0.0001 |
| **Hypertension** |  |  |
| Yes | Ref. |  |
| No | 58.8 (39.9, 77.6) | <0.0001 |
| **Diabetes** |  |  |
| Yes | Ref. |  |
| No | 83.2 (51.9, 114.6) | <0.0001 |
| Borderline | 12.3 (-50.3, 75.0) | 0.7010 |
| **Sleep disorders** |  |  |
| Yes | Ref. |  |
| No | 47.7 (20.3, 75.2) | 0.0014 |

Abbreviations: RIP, ratio of family income to poverty; BMI, body mass index; HDL, high-density lipoprotein; LDL, low-density lipoprotein; HOMA-IR, homeostasis model assessment of insulin resistance; TyG, triglyceride glucose index; TyG-WC, triglyceride glucose-waist circumference
